# Risk of adverse fetal outcomes following nonobstetric surgery during gestation: a nationwide population-based analysis

**DOI:** 10.1186/s12884-022-04732-w

**Published:** 2022-05-13

**Authors:** Pei-Han Fu, Chia-Hung Yu, Yi-Chen Chen, Chin-Chen Chu, Jen-Yin Chen, Fu-Wen Liang

**Affiliations:** 1 Department of Anesthesiology, Chi Mei Medical Center, Tainan, Taiwan; 2 Department of Medical Research, Chi Mei Medical Center, Tainan, Taiwan; 3 Department of Public Health, College of Health Sciences, Kaohsiung Medical University, Kaohsiung, Taiwan; 4 Department of Medical Research, Kaohsiung Medical University Hospital, Kaohsiung Medical University, Kaohsiung, Taiwan; 5 Center for Big Data Research, Kaohsiung Medical University, Kaohsiung, Taiwan

**Keywords:** Nonobstetric surgery, Fetal outcomes, Neonatal outcomes

## Abstract

**Background:**

Literature suggests that nonobstetric surgery during gestation is associated with a higher risk of spontaneous abortion, prematurity, and a higher cesarean section rate, but the direct impact on fetal outcomes is still unclear. In this study, we aimed to investigate whether nonobstetric surgery during pregnancy is associated with negative fetal outcomes by analysing a nation-wide database in Taiwan.

**Methods:**

This population-based retrospective observational case–control study was based on the linkage of Taiwan’s National Health Insurance Research Database, Birth Reporting Database, and Maternal and Child Health Database between 2004 and 2014. For every pregnancy with nonobstetric surgery during gestation, four controls were randomly matched according to maternal age and delivery year. We estimated adjusted odds ratios (aOR) and 95% confidence intervals (CIs) of adverse fetal outcomes with the non-surgery group as the reference. The primary outcomes involved stillbirth, prematurity, low birth weight, low Apgar scores, and neonatal and infant death.

**Results:**

Among 23,721 identified pregnancies, 4,747 underwent nonobstetric surgery. Pregnancies with nonobstetric surgery had significantly higher risks of prematurity (aOR: 1.46; 95% CI: 1.31–1.62), lower birth weight (aOR: 1.49; 95% CI: 1.33–1.67), Apgar scores < 7 (1 min, aOR: 1.58; 95% CI: 1.33–1.86; 5 min, aOR: 1.34; 95% CI: 1.03–1.74), neonatal death (aOR: 2.01; 95% CI: 1.18–3.42), and infant death (aOR: 1.69; 95% CI: 1.12–2.54) than those without nonobstetric surgery after adjustment for socioeconomic deprivation, hospital level, and other comorbidities. Surgery performed in the third trimester was associated with a significantly increased rate of prematurity (aOR: 1.38; 95% CI: 1.03–1.85), but lower rates of stillbirth (aOR: 0.1; 95% CI: 0.01–0.75) and Apgar score < 7 at the 5^th^ minute (aOR: 0.2; 95% CI: 0.05–0.82), than surgery performed in the first trimester.

**Conclusions:**

Pregnancies with nonobstetric surgery during gestation were associated with increased risks of prematurity, low birth weight, low Apgar scores, neonatal and infant death, longer admission, and higher medical expenses than those without surgery. Furthermore, surgery in the third trimester was associated with a higher rate of prematurity than surgery performed in the first trimester.

**Trial registration:**

Not applicable.

## Background

Nonobstetric surgery during gestation is a relatively rare clinical scenario. The prevalence of nonobstetric surgery during gestation is approximately 0.75%–2.0% [1]. Abdominal surgery is the most common, followed by surgeries on dentition, nail skin, and bone [2]. The decision to perform surgery is a difficult one to make, given the potential risks for the fetus and complicated surgical conditions due to the gravid uterus. Since nonobstetric surgery may be required in any trimester, it is imperative to determine whether the surgery will affect the fetus in any manner.

The American College of Obstetricians and Gynecologists Committee on Obstetric Practice suggests that an emergent surgery should never be denied or delayed regardless of the trimester, while elective surgery could be postponed until after delivery [3]. Nevertheless, there are grey areas when the surgery is necessary but not emergent, such as an open reduction internal fixation surgery for bone fracture. Whether the trimester during which the surgery is performed really matters remains unclear, as well as the impact of the trimester. These scenarios require cautious evaluation of the benefit and potential risk, yet there is little evidence in the context of fetal outcomes.

Most previous studies focused on maternal surrogate outcomes rather than direct fetal outcomes, which stems from the difficulty of linking mothers to neonates and missing information of the offspring [4–6]. Some studies on perinatal conditions reported that nonobstetric surgery increased the risks of miscarriage, preterm labour, and low birth weight [7–9]. However, there is still a lack of detailed information about fetal conditions. In this study, we aimed to investigate whether nonobstetric surgery during pregnancy is associated with adverse fetal outcomes using a nationwide population-based database in Taiwan. In addition to similar outcomes mentioned in previous literature, we addressed detailed fetal condition, including Apgar scores and survival in the neonatal and infant stage. Furthermore, we would clarify and compare the influences of nonobstetric surgery on the fetus among different trimesters.

## Methods

### Data source

We extracted data from Taiwan’s National Health Insurance Research Database (NHIRD), Birth Reporting Database (BRD), and the Taiwan Maternal and Child Health Database (TMCHD) between 2004 and 2014, which were provided by the Ministry of Health and Welfare. Taiwan’s National Health Insurance (NHI) program has been implemented since 1995 and covers 99% of the Taiwanese population. The NHIRD contains the patient’s date of birth, socioeconomic status, the ICD-9-CM (International Classification of Diseases, Ninth Revision, Clinical Modification) codes of diagnoses and procedures, dates of admission and discharge, and costs covered by the NHI. The BRD collects all live births and stillbirths with weight > 500 g or gestational age > 20 weeks delivered in Taiwan. This dataset provides information regarding birth weight, gestational age, delivery method, Apgar scores, and maternal age. The TMCHD contains the encrypted national identification numbers of children and their parents, which provide the parent–child linkages. In our study, we used it to interlink the medical claims of offspring and their mothers. All identifiers were encrypted before data released by the Ministry of Health and Welfare to ensure privacy [10].

### Study sample

**Fig. 1:**
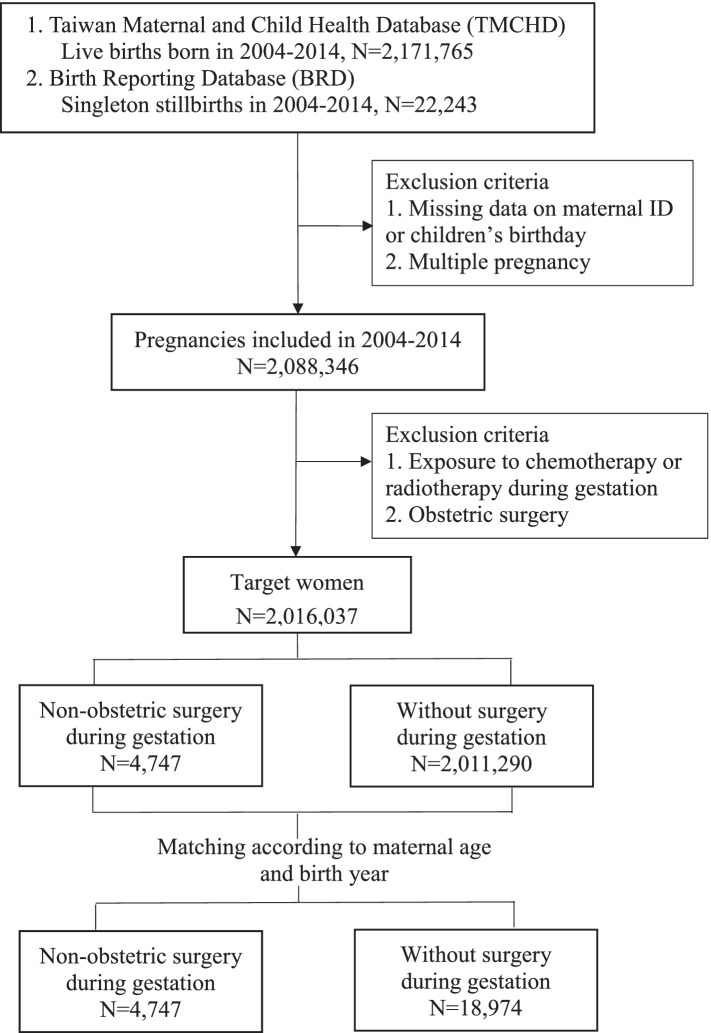
Flow chart showing the process of selecting the study sample

We collected delivery events between 2004 and 2014 to identify parturient women from the NHIRD; subsequently, we traced back the gestation period to determine whether the individual had undergone nonobstetric surgery under general or regional anaesthesia (Fig. 1). Nonobstetric surgery was defined as any surgery recorded during pregnancy except for obstetric surgery (ICD-9 procedure codes 72–75). Moreover, we excluded parturient women who accepted cervical cerclage (ICD-9 procedure codes 67.5–67.6) for threatened abortion given the speciality of the condition, which should be individually discussed. Additionally, we excluded patients who underwent radiotherapy or chemotherapy during pregnancy or multiple surgeries during one gestation, or had multiple pregnancies. From 2004 to 2014, 2,088,346 women had singleton deliveries. After excluding teratogenic factors, including chemotherapy/radiotherapy, obstetric surgery, and cervical cerclage, we included 4747 pregnancies with nonobstetric surgery during gestation. For each pregnancy with nonobstetric surgery, we included four random controls matched according to maternal age and delivery year.

Patients were classified according to age into five categories: 18–24, 25–29, 30–34, 35–39, and ≥ 40 years. Certain pre-existing comorbidities were determined from medical claims records within 1 year before surgery, including hypertension (ICD-9-CM codes 401–405, 997.91, 642.0–642.3, 642.9), diabetes mellitus (ICD-9-CM codes 249, 250, 648.0, 648.8), cardiac disease (ICD-9-CM codes 391, 393–398, 410–416, 420–429), chronic renal disease (ICD-9-CM codes 403, 582, 583, 585, 586, 588, 646.2), thyroid disease (ICD-9-CM codes 240–246), previous caesarean section (ICD-9-CM code 654.2), and preeclampsia or eclampsia (ICD-9-CM codes 642.4–642.7).

Additionally, we considered the location of deliveries. Based on the definition of the Taiwan Joint Committee on Hospital Accreditation, a local hospital refers to a hospital with < 300 beds and only provides primary medical services. Regional hospitals have 301–999 beds and provide secondary medical services. Medical centres have 1,000–2,500 beds and are responsible for most of the staff training burden, provide tertiary medical services, and possess research facilities.

### Outcomes

The primary outcomes were stillbirth, prematurity, low birth weight, low Apgar scores, and neonatal and infant death. Neonatal and infant deaths were defined as death occurring within 28 days and 1 year, respectively, after birth. The secondary outcomes were fetal admission length and medical expenses.

### Statistical analysis

Categorical and continuous variables are presented as number (percentile) and median (interquartile range), respectively. Between-group differences in baseline characteristics and comorbid variables were evaluated using Pearson’s χ^2^ test and the Wilcoxon rank-sum test for categorical and continuous variables, respectively. Multivariable logistic regression analyses were performed for between-group assessments of the adjusted odds ratio (aOR) with 95% confidence interval (CI) of adverse fetal outcomes after adjusting for socioeconomic status, hospital level, and comorbidities. Statistical analyses were performed using SAS version 9.4 (SAS Institute, Cary, NC, USA). Statistical significance was set at *p* < 0.05.

## Results

We included 4,747 pregnancies with nonobstetric surgery between 2004 and 2014. Regarding 4:1 matching for age and delivery year, we retrieved 18,974 randomly selected controls for the non-surgery group after excluding missing data (*n* = 14). Table 1 presents the maternal demographic characteristics of the study groups. Among women with nonobstetric surgery during pregnancy, 2,163 (45.57%) underwent operations in the first trimester, 2,059 (43.37%) in the second, and 525 (11.06%) in the third trimester.

**Table 1:** Demographic characteristics of pregnant women with and without nonobstetric surgery during gestation in Taiwan from 2004 to 2014

	**Nonobstetric surgery during gestation**		
	**Without**	**With**	
Overall, N	18,974	4,747	
Variable	N (%)	N (%)	*P*-value^c^
Maternal age (years)			
18–24	2724 (14.4)	681 (14.4)	1.0000
25–29	5603 (29.5)	1401 (29.5)	
30–34	6749 (35.6)	1689 (35.6)	
35–39	3287 (17.3)	823 (17.3)	
≥ 40	611 (3.2)	153 (3.2)	
Socioeconomic deprivation, missing (*N* = 364)	270 (1.5)	98 (2.1)	0.0022
Comorbidities			
Hypertension^a^	428 (2.3)	182 (3.8)	< 0.0001
Diabetes mellitus^b^	2349 (12.4)	665 (14.0)	0.0026
Cardiac disease	340 (1.8)	142 (3.0)	< 0.0001
Chronic renal disease	37 (0.2)	42 (0.9)	< 0.0001
Thyroid disease	507 (2.7)	214 (4.5)	< 0.0001
Obstetric condition			
Previous caesarean section	2731 (14.4)	728 (15.3)	0.9980
Preeclampsia/Eclampsia	350 (1.8)	131 (2.8)	< 0.0001
Hospital level, missing (*N* = 512)			
Medical centre	3281 (17.7)	1186 (25.7)	< 0.0001
Non-medical centre	15,693 (82.3)	3561 (74.3)	
Trimesters			
1^st^	NA	2163 (45.57)	
2^nd^	NA	2059 (43.37)	
3^rd^	NA	525 (11.06)	

^a^ Hypertension was defined as hypertension or gestational hypertension

^b^ Diabetes mellitus was defined as diabetes or gestational diabetes

^c^ Pearson’s chi-square test

We further examined whether nonobstetric surgery was associated with adverse fetal outcomes (Table 2). Compared to those in the group without surgery, babies of mothers undergoing nonobstetric surgery were associated with higher risks of prematurity (aOR: 1.46; 95% CI: 1.31–1.62), low birth weight (aOR: 1.49; 95% CI: 1.33–1.67), Apgar scores < 7 at the 1^st^ minute (aOR: 1.58; 95% CI: 1.33–1.86) and 5^th^ minute (aOR: 1.34; 95% CI: 1.03–1.74), neonatal death (aOR: 2.01; 95% CI: 1.18–3.42), and infant death (aOR: 1.69; 95% CI: 1.12–2.54) after adjustment for socioeconomic deprivation, comorbidities, and hospital level. Compared to the group without surgery, the nonobstetric surgery group showed significantly longer admission days (6 vs. 5 days, *p* = 0.0001) and higher medical expenses within 30 days (US$821 vs. US$682, *p* < 0.0001, *p* < 0.0001) after birth.

**Table 2:** The adverse fetal outcomes following nonobstetric surgery during gestation

	**Nonobstetric surgery during gestation**			**Nonobstetric surgery, trimester**			
	**Without**	**With**	**With vs. without**	**1**^**st**^	**2**^**nd**^	**3**^**rd**^	
**Overall, N**	**18,974**	**4,747**		**2,163**	**2,059**	**252**	
**Outcome**	**N (%)**	**N (%)**	**aOR**^**c**^** (95% CI)**	**N (%)**	**N (%)**	**N (%)**	***P*-value**
**Stillbirth**	241 (1.3)	118 (2.5)	1.28 (0.97–1.70)	53 (2.5)	62 (3.0)	3 (0.6)	< 0.0001
**Prematurity**	1540 (8.1)	632 (13.3)	1.46 (1.31–1.62)	259 (12.0)	288 (14.0)	85 (16.2)	0.0189
**Low birth weight**	1364 (7.2)	571 (12.0)	1.49 (1.33–1.67)	246 (11.4)	250 (12.1)	75 (14.3)	0.1799
**Apgar scores**, missing (*N* = 142)							
1 min < 7	541 (2.9)	251 (5.4)	1.58 (1.33–1.86)	104 (4.9)	124 (6.1)	23 (4.4)	0.1192
5 min < 7	256 (1.4)	109 (2.3)	1.34 (1.03–1.74)	51 (2.4)	55 (2.7)	3 (0.6)	0.0148
**Neonatal death**	45 (0.2)	26 (0.55)	2.01 (1.18–3.42)	11 (0.51)	13 (0.63)	2 (0.38)	0.7432
**Infant death**	81 (0.4)	40 (0.84)	1.69 (1.12–2.54)	18 (0.8)	19 (0.9)	3 (0.6)	0.0218
**Healthcare utilization within 30 days after birth**							
Median admission days (IQR)^a^	4 (5)	5 (6)	NA	4 (5)	5 (6)	5 (8)	0.0527
Median cost, US dollar^b^ (IQR)^a^	682(1187)	821(1525)	NA	771(1437)	831(1741)	844(2032)	0.2151

^a^ IQR stands for interquartile range, which is the difference between the 75^th^ percentile and the 25^th^ percentile

^b^ 1 US dollar = 28.9 Taiwan dollars

^c^ adjustment for socioeconomic deprivation, comorbidities, and hospital level

**Fig. 2:**
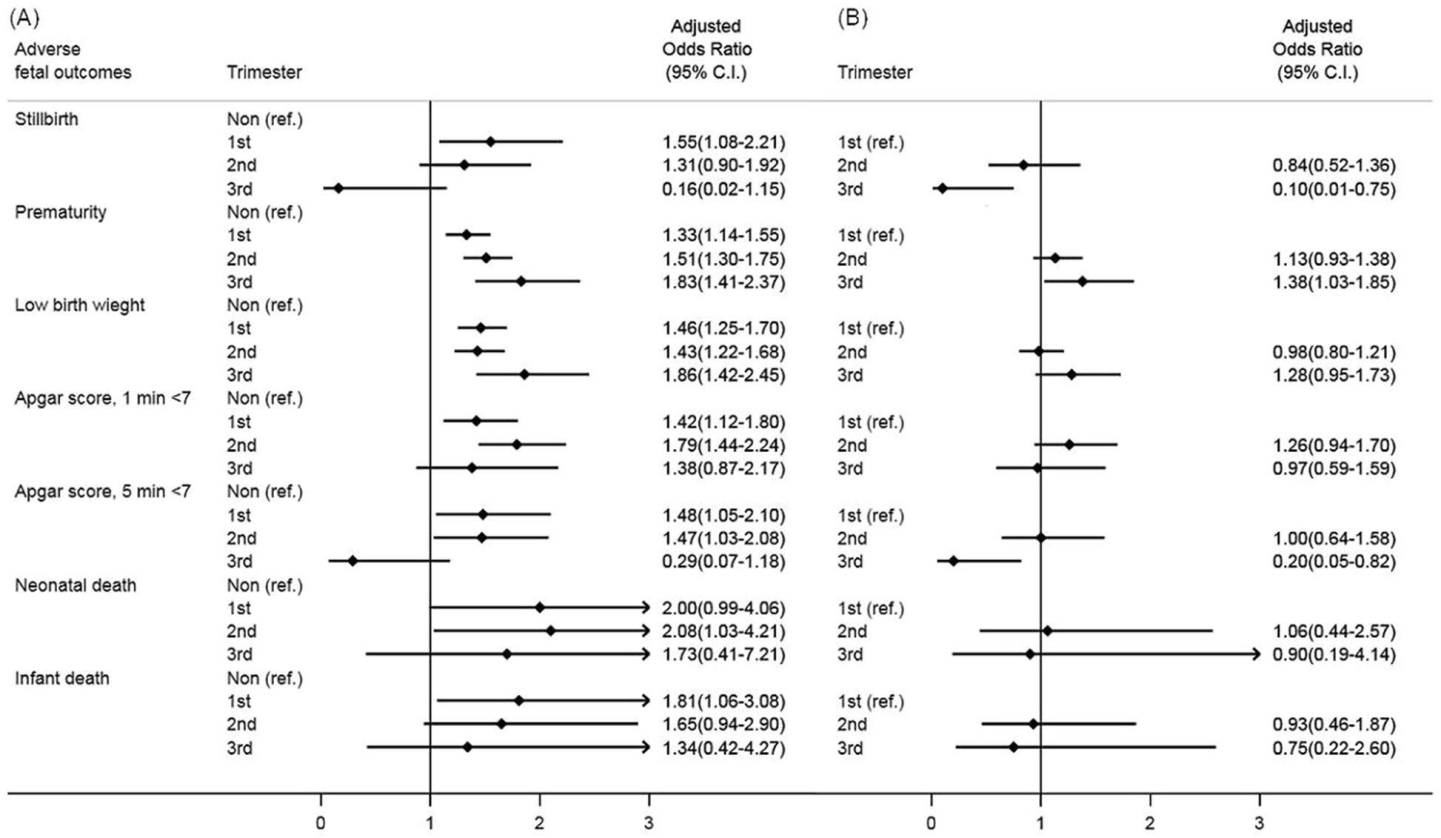
Forest plots for relative risks of adverse fetal outcomes following nonobstetric surgery performed in different trimesters with (**A**) non-surgery group as the reference, and (**B**) surgery performed in the first trimester as the reference group

We further analysed the risk of adverse fetal outcomes among women undergoing nonobstetric surgery in different trimesters (Table 2). The proportions of stillbirth, prematurity, Apgar score < 7 at 5^th^ minute, and infant death were significantly different across three trimesters. Subsequently, we estimated the aOR (95% CI) with the non-surgical group as a reference, after controlling for socioeconomic status, comorbidities, and hospital level (Fig. 2A). The group with nonobstetric surgery showed significantly higher risks of prematurity and low birth weight in all trimesters. Regarding Apgar scores < 7 at the 1^st^ and 5^th^ minute, the risks were significantly increased when nonobstetric surgery performed in the first and second trimesters. Nonobstetric surgery in the first trimester was also associated with a higher risk of stillbirth (aOR, 1.55; 95% CI, 1.08–2.21) and infant death (aOR, 1.81; 95% CI, 1.06–3.08) than no surgery. Further, nonobstetric surgery in the second trimester was associated with an increased risk of neonatal death (aOR, 2.08; 95% CI, 1.03–4.21).

Then, we examined the odds of adverse fetal outcomes across different trimesters with the surgery performed in the first trimester as a reference (Fig. 2B). After adjustment for socioeconomic status, comorbidities, and hospital level, surgery performed in the third trimester was associated a significantly higher risk of prematurity (aOR, 1.38; 95% CI, 1.03–1.85), but a lower risk of stillbirth (aOR, 0.10; 95% CI, 0.01–0.75) and Apgar scores < 7 at the 5^th^ minute (aOR, 0.20; 95% CI, 0.05–0.82), compared with those in the first trimester.

## Discussion

### Main findings

We observed higher risks of prematurity, low birth weight, Apgar scores < 7 at the 1^st^ minute and 5^th^ minute, neonatal death, and infant death after nonobstetric surgery during pregnancy compared with pregnancies without surgery, after adjustment for socioeconomic deprivation, comorbidities, and hospital level. The surgery group showed longer fetal admissions and higher medical expenses than the non-surgery group. Furthermore, surgery performed in the third trimester was associated with a significantly increased rate of prematurity, but lower rates of stillbirth and Apgar score < 7 at the 5th minute, than surgery performed in the first trimester.

### Strengths and limitations

The major strength of this population-based case–control study is the usage of the linkable nationwide database, which included most pregnancies and fetal information of the whole population in Taiwan between 2004 and 2014. In addition to maternal outcomes, we addressed some detailed perinatal outcomes including Apgar scores, neonatal and infant death, fetal admission length, and medical expenses. Furthermore, we clarified and compared the relative risks of adverse fetal outcomes following nonobstetric surgery among different trimesters. However, some limitations should be considered while interpreting the findings. First, a major concern regarding claim database analysis is the accuracy of the records. Despite the possibility of some incorrect ICD-9 coding, most codes were reviewed and validated by auditors to ensure the accuracy of the claims. Some cases with incomplete or aberrant data were excluded at the beginning of the study. Second, the inherent limitations of an observational study should be considered. We could only adjust for potential confounders recorded in the database, which did not include some information such as number of parity or gravidity, body mass index, the severity of comorbidities, and lifestyle behaviours. Since nonobstetric surgery is a rare event with a relatively small sample size, including too many unnecessary variables may cause overfitting or involving collinearity. As a result, we chose strong confounders scientifically to ensure the power and generalizability of the results. Third, the findings of this nationwide population-based study cannot be generalised to the populations of other countries due to the differences in race, culture, and medical resources. Finally, this study was not stratified according to disparate types of surgery because of the sheer variety in the types of surgery conducted. Rough categorization, such as according to surgical site or functional system, would result in considerable heterogeneity within each category; meanwhile, too many categories would cause insufficient power, not to mention difficulty in matching according to surgery and surgical criteria. Therefore, it would be better to discuss and compare individual surgery in a separate project. Nevertheless, this study provided a fairly clear picture of the relationship between negative fetal outcomes, the incidence and relative risk, and nonobstetric surgery during gestation in Taiwan over 10 years. The results and association should be considered in the decision making of elective surgery for pregnant women and their healthcare providers.

### Interpretation

Nonobstetric surgery during pregnancy has long been a concern for women and healthcare providers, particularly the potential risk of negative influences on the fetus. In our study, most operations were performed in the first trimester (45.57%), followed by the second (43.37%) and third trimesters (11.06%). On the contrary, a large-scale study in the United Kingdom reported that 45%, 26%, and 29% of surgeries were conducted in the first, second, and third trimesters, respectively [3].

Considering ethical issues, there are few randomised clinical trials in pregnant women, particularly when it comes to the causation effect of nonobstetric surgery on adverse outcomes. Several retrospective studies had investigated the association between surgical intervention and pregnancy outcomes. In 2005, Cohen et al. reported an increased risk of fetal loss upon occurrence of peritonitis [6]. A large scale study in the United Kingdom used National Health Service data to estimate the risk of adverse birth outcomes in pregnant women undergoing nonobstetric surgery and observed an increased risk of miscarriage, preterm labour, low birth weight, and caesarean section [7]. Another nationwide sampling case–control study reported increased rates of spontaneous abortion, caesarean section rate, and preterm labour following nonobstetric surgery during gestation [5]. A Danish registry-based cohort study reported that nonobstetric abdominal surgery during pregnancy was associated with an increased risk of adverse birth outcomes, including small for gestational age, preterm labour, and miscarriage [8].

Our study aimed to perform a detailed investigation into the outcomes of the offspring born after such pregnancies. We observed an association between nonobstetric surgical intervention during pregnancy and higher risks of stillbirth, prematurity, and low birth weight after adjusting for socioeconomic status, comorbidities, and hospital level, which is consistent with previous findings [7]. Furthermore, we have some novel findings that the surgery group was related to higher rates of poor Apgar score, neonatal death, infant death, longer neonatal admission, and higher medical expenses than the group without surgery, which could also be partly attributed to increased rates of prematurity and low birth weight [11, 12].

When nonobstetric surgery was performed, those conducted in the third trimester were associated with a higher risk of preterm delivery than those conducted in the first trimester in both our study and the previous studies [5, 7, 13, 14]. To the best of our knowledge, there is no evidence regarding the teratogenic effects of nonobstetric surgery and anaesthetic agents, even during early pregnancy [3, 6, 15]. However, an association between increased rate of stillbirth and infant death and surgery performed in the first trimester compared to that performed in the third trimester was revealed in this study. While higher rates of low Apgar scores were noted in pregnancies with surgery performed in early period of pregnancy, those with surgery in the third trimester showed no significant difference compared to the non-surgery group. It seems that a more matured fetus could be more resilient to adverse outcomes including poor Apgar scores and stillbirth, but not prematurity and low birth weight, which might be related to an irritated uterus [16]. However, there was no adequate power to rule out false negative for surgery in the third trimester, which might fail to illuminate the true relationships. Large-scale prospective studies might be required to clarify this association further.

## Conclusion

Nonobstetric surgery during gestation was associated with higher rates of prematurity, low birth weight, low Apgar scores, and neonatal and infant death, as well as longer fetal admission and higher medical expenses, compared with the non-surgery group. Furthermore, nonobstetric surgery performed in the third trimester was associated with a higher risk of prematurity but lower rate of poor Apgar scores and stillbirth than surgery performed in the first trimester. Since this is a retrospective observational study, our findings suggest an association rather than causation. Nevertheless, our findings depict a contour of the relationship between nonobstetric surgery during gestation and adverse fetal outcomes, which could provide further information for decision making among clinical practitioners and pregnant women, especially in the context of elective surgery. Due to ethical issues, it is impractical to expect a large-scale randomised clinical trial in pregnant women for confirming the causation effect of nonobstetric surgery on adverse outcomes. Further large-scale prospective studies could help to infer the causal relationship between surgical interventions during pregnancy and adverse fetal outcomes. In addition, further research is warranted to stratify according to or focus on individual surgery, which could help to identify risky surgeries in pregnant women.

## Data Availability

The data that support the findings of this study are available from the Collaboration Center of Health Information Application, but restrictions apply to the availability of these data, which were used under license for the current study, and so are not publicly available. Data are however available from the authors upon reasonable request and with permission of the Ministry of Health and Welfare.

## Abbreviations

NHIRD: Taiwan’s National Health Insurance Research Database
BRD: Birth Reporting Database
TMCHD: Taiwan Maternal and Child Health Database
NHI: National Health Insurance
ICD-9-CM: International Classification of Diseases, Ninth Revision, Clinical Modification
aOR: Adjusted odds ratio
CI: Confidence interval
